# Changes in cosmic radiation doses of aircraft crew over the COVID-19 pandemic (2019–2022)

**DOI:** 10.3389/fpubh.2025.1674819

**Published:** 2025-10-31

**Authors:** Hiroshi Yasuda, Kazuaki Yajima

**Affiliations:** ^1^Research Institute for Radiation Biology and Medicine (RIRBM), Hiroshima University, Hiroshima, Japan; ^2^National Institutes for Quantum Science and Technology, Chiba, Japan

**Keywords:** cosmic radiation, aircraft crew, aviation dose, COVID-19 pandemic, JISCARD

## Abstract

The in-flight cosmic radiation exposure of crew members in commercial jet aircraft is considered occupational exposure. In Japan, a guideline for the management of in-flight exposure of aircraft crews was established in 2006 by the radiation council. Accordingly, in-flight doses of aircraft crews have been calculated, and their annual in-flight doses have been recorded for the last 18 years, for which the authors have cooperated with airlines to calculate aviation route doses on a monthly basis. In this brief report, we present the trend of annual dose distributions of cabin attendants (CAs) and pilots (PLs) working in one of the Japanese airlines over the period of the COVID-19 pandemic from fiscal year 2019 to 2022. The cosmic radiation exposure of the CAs was significantly affected by the pandemic. The percentage of the CAs who annually received >1 mSv sharply decreased from 65% in the pre-pandemic year (2019) to 4.6% in the first year of the pandemic (2020), and their collective doses notably decreased in 2020 to 30% of the pre-pandemic level, followed by gradual recovery in subsequent two years. In contrast, the annual dose distribution of the PLs did not show a notable change, which is attributable to the increase in cargo flights and the introduction of small aircrafts during the pandemic.

## 1 Introduction

An increase in certain types of cancers, such as skin and breast cancers, has been observed in aircraft crews although a causal relationship is yet to be clarified (1–4). The enhanced cosmic radiation at aviation altitude is considered to be one of the causes of the cancer risk increase, as the effective dose rate of cosmic radiation at typical cruising altitude (10–12 km) is approximately 100 times higher than that on the ground. At aviation altitudes, neutrons account for approximately 50% of the effective dose, with protons contributing 10–20%, whereas the muons account for nearly 80% dose on the ground (5). Based on these facts, the International Commission on Radiological Protection (ICRP) recommends that the exposure of personnel to cosmic radiation during the operation of commercial jet aircraft should be treated as occupational exposure (6–8).

Along the ICRP recommendations and corresponding legal settings, in-flight cosmic radiation exposure of aircraft crews has been managed in selected countries or regions, such as the United States (9), Europe (10–12), and Japan (13). Partial data on the cosmic radiation doses of aircraft crews indicated that their annual doses were 1–3 mSv on average and 4–6 mSv in maximum (13–17). For example, according to an only source that were published by the Japanese government, the annual in-flight effective doses of the aircraft crews of Japanese airlines operating international flights were approximately 2 mSv on average and 4 mSv at maximum, as shown in Table 1 (13). Considering the reality of the cosmic radiation exposure of aircraft crews, national authorities have set regulations or guidance with reference dose levels above which additional protective actions, such as more precise individual monitoring and longer record keeping, need to be taken. The current reference levels are 6 mSv y^−1^ in most European countries (10–12) and 5 mSv y^−1^ in Japan (13).

**Table 1:** Annual in-flight effective doses of aircraft crews of Japanese airlines operating international flights in the fiscal year 2007 (April 1, 2007 to March 31, 2008) (13).

	Cabin attendants (CA)	Pilots (PL)
Number	12,219	5,851
Maximum effective dose [mSv]	4.24	3.79
Mean effective dose [mSv]	2.15	1.68

However, these data were published before the COVID-19 pandemic began in early 2020, and its effects on the cosmic radiation exposure of aircraft crews remain unclear. During the initial phase of the pandemic, people feared the serious consequences of infection with severe acute respiratory syndrome coronavirus 2 (SARS-CoV-2), and many of them cancelled air travel. Subsequent international and domestic travel restrictions and quarantine regulations caused a remarkable drop in flights on a global scale (18–20), which is presumed to have significantly reduced the cosmic radiation exposure of aircraft crews, as reported for Japanese passengers (21). Therefore, in this study, we present the trend of in-flight dose distributions of aircraft crew working for a Japanese airline over the period of the COVID-19 pandemic (2019–2022), with the aim of contributing to discussions about the total impact of this pandemic on public and occupational health.

## 2 Methods

This study focused on the four-years period over the COVID-19 pandemic from 2019 (one year before the pandemic) to 2022 (third year of the pandemic). Annual in-flight doses of aircraft crews for this period were provided by a Japanese airline without traceable information that could be used to identify individuals. The dose from cosmic radiation during each flight (hereafter called “route dose”) was calculated based on a flight plan, which was determined by the airline. The authors cooperated in the route-dose calculations using the program “JISCARD EX” that was developed as part of the program package “JISCARD” (22) for the management of cosmic radiation exposure of aircraft crews in Japan (13). JISCARD EX incorporated an analytical code for calculation of atmospheric dose rates, named ‘PARMA,’ which was originally developed in Japan (23) and, by default, employed the up-to-date radiation and tissue weighting factors recommended in the ICRP recommendations (7). The accuracy of the aviation doses calculated by the JISCARD EX was validated through comparisons with in-flight measurements (24–27) and different calculation codes developed in other countries (28, 29). In the comparison with German code EPCARD. Net, the route doses calculated by JISCARD EX for 68 major commercial flight routes agreed within ±20%, which is considered satisfactory for radiological protection purposes (28).

Route doses calculated by the JISCARD EX were provided to the airline every month. The airline estimated the individual effective doses of aircraft crews for each fiscal year (April–March) by adding the route doses provided by the authors and their flight records during the respective period. In this process, the route doses of domestic flights were uniformly given as 2 μSv per flight after careful examination of the dose levels and their uncertainties for the major domestic routes. For examples, we confirmed that the route doses from Tokyo/Haneda to Chitose (flight time: 70 min) were approximately 1.9 μSv in 2019 and 1.3 μSv in 2022, and those from Tokyo/Haneda to Fukuoka (flight time: 90 min) were approximately 2.6 μSv in 2019 and 2.4 μSv in 2022; the flying times of most other domestic flights were between those of these two routes. As the levels of any domestic route doses in Japan were significantly lower than those of long-haul international flights from Japan to Europe or North America (>50 μSv) (21), it was considered that the impact of this simplification was small and acceptable from the viewpoint of radiological protection. Therefore, we judged that a uniform route dose of 2 μSv per domestic flight could be rationalized for the management of cosmic radiation exposure of Japanese aircraft crews.

## 3 Results

**Figure 1:**
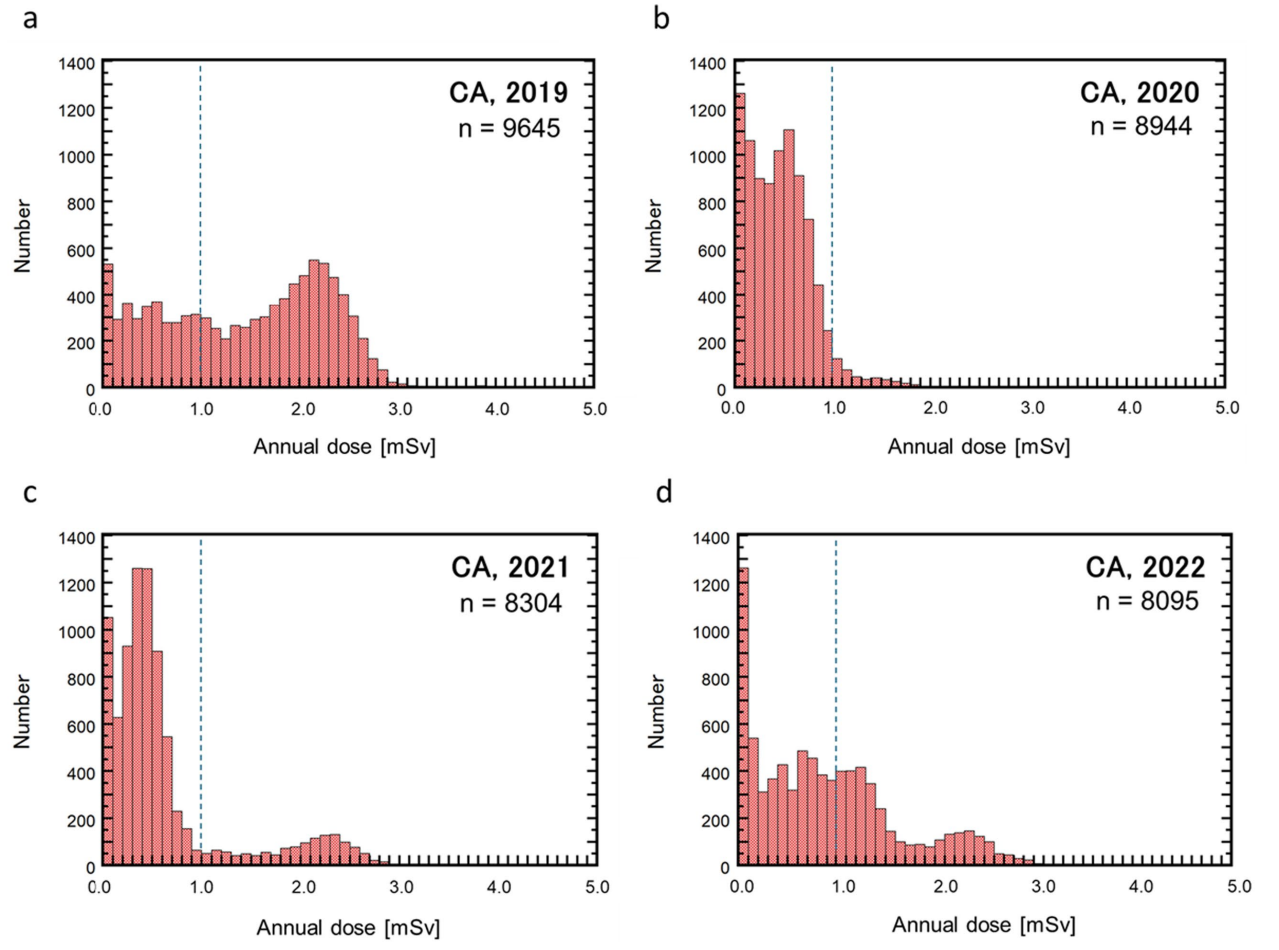
Histograms showing annual effective doses of cabin attendants (CA) for fiscal years 2019 **(a)**, 2020 **(b)**, 2021 **(c)**, and 2022 **(d)**. A vertical dashed line in each graph marks the level of 1.0 mSv.

The histograms of the annual effective doses of cabin attendants (CA) for each fiscal year are shown in Figure 1 where the dose level of 1 mSv is indicated with a dotted line in each graph, considering that the European Union directive requires European airlines whose aircraft crew may annually receive an effective dose greater than 1 mSv to carry out dose assessments (10–12). Remarkable changes in the dose levels were observed during the focused period (2019–2022). The maximum and mean CA doses in 2020 were 2.7 mSv and 0.5 mSv, respectively, both of which were much smaller than those in 2019 (4.8 mSv and 1.4 mSv, respectively) and also those in 2007 (4.2 mSv and 2.2 mSv, respectively) shown in Table 1. The dose levels gradually recovered in subsequent years with increasing mean dose (0.6 mSv in 2021 and 0.9 mSv in 2022). It should be noted that, while the total number of CA did not notably change during the pandemic period, the percentage of the CAs who received the annual doses of >1 mSv remarkably decreased from 65% in 2019 to 5% in 2020, followed by a gradual increase to 15% in 2021 and 40% in 2022. As a result, the collective dose decreased by 29% in 2020 and recovered to approximately 50% by 2022, compared to the 2019 values. The bimodal shapes of the CA dose distributions can be attributed to the flight regions they mainly engaged in; the crew engaged in flights to Asian or Oceanian cities from Japan, including domestic flights, receive notably lower doses than those engaged in long-haul flights to European or North American cities.

**Figure 2:**
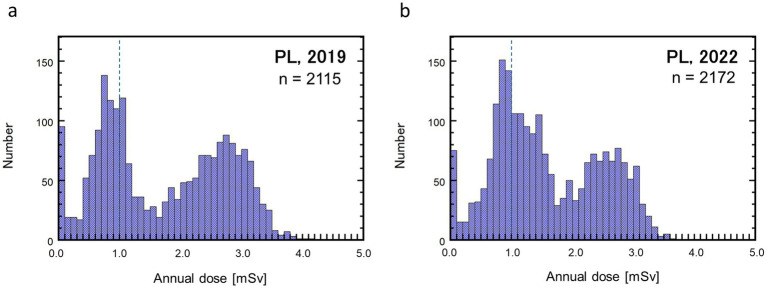
Histograms showing annual effective dose distributions of pilots (PL) for 2019 **(a)** and 2022 **(b)**. A vertical dashed line in each graph marks the level of 1.0 mSv.

Histograms of the annual doses of pilots (PL) are shown in Figure 2. Unfortunately, the annual doses of PL in the initial period of the pandemic (2020–2021) were missing because the unexpected changes in their work plan caused the boarding of flights that did not have monthly flight plans, which were necessary for calculating route doses. Nevertheless, it was confirmed that the change in PL doses between the pre-pandemic year (2019) and the third year of the pandemic (2022) was much smaller than that in CA doses. The PL dose distributions in both years exhibited similar bimodal shapes, which could be deconvoluted into two-peak normal distributions. The maximum and mean PL doses in 2020 were 4.1 mSv and 1.7 mSv, respectively, while those in 2022 were 3.7 mSv and 1.6 mSv, respectively. These dose values were comparable to those in 2007 (3.8 mSv and 1.7 mSv, respectively) (Table 1). Unlike the CA doses, the percentage of the PLs who received >1 mSv slightly increased from 65% in 2019 to 68% in 2022, whereas the collective dose decreased by approximately 5%.

**Figure 3:**
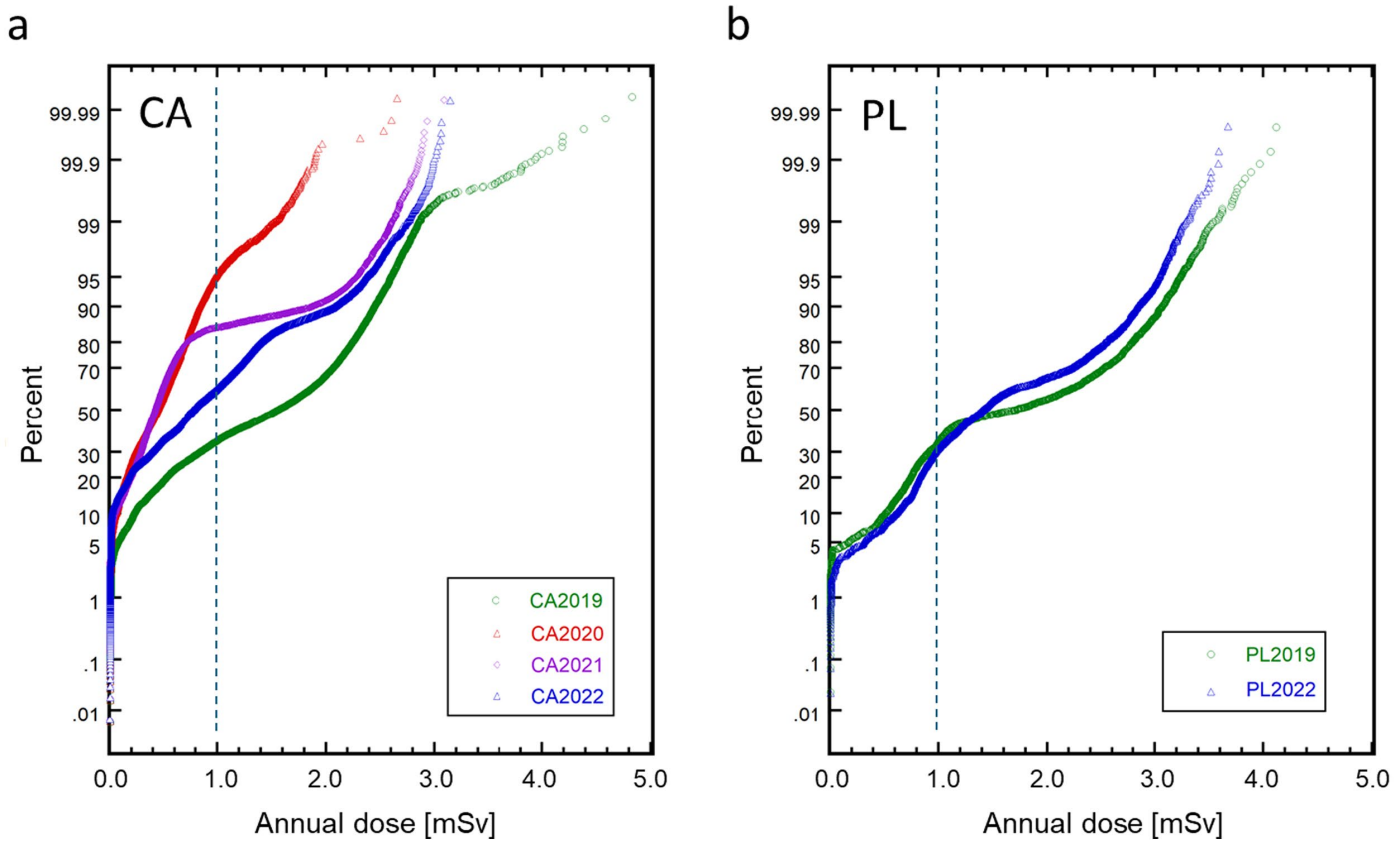
Cumulative probability plots of annual effective doses of cabin attendants (CA) **(a)** and pilots (PL) **(b)** from fiscal year 2019 to 2022. A vertical dashed line in each graph marks the level of 1.0 mSv.

Figure 3 shows the cumulative probability plots of the annual doses of CAs (Figure 3A) and PLs (Figure 3B) for the study period. The CA doses exhibited remarkable changes in both dose level and distribution shape with time. After an overall notable reduction in the first year of the pandemic (2020) from the previous year, a prompt recovery of the CA doses in 2021 was observed only in the higher dose range, followed by considerable recovery in the lower dose range in 2022. In contrast, the PL doses did not show a notable change between the pre-pandemic year (2019) and the third year of the pandemic (2022) (Figure 2). The selected statistical values regarding the annual doses of CAs and PLs are summarized in Table 2.

**Table 2:** Statistical values on the annual in-flight effective doses of aircraft crews of a Japanese airline over the COVID-19 pandemic period (fiscal year 2019–2022).

	Cabin attendants (CA)				Pilots (PL)	
	2019	2020	2021	2022	2019	2022
Number	9,645	8,944	8,304	8,095	2,115	2,172
Minimum effective dose [mSv]	0.004	0.004	0.002	0.002	0.004	0.006
Maximum effective dose [mSv]	4.826	2.656	3.088	3.146	4.118	3.674
Mean effective dose [mSv]	1.440	0.455	0.616	0.889	1.726	1.590
Median dose [mSv]	1.560	0.439	0.423	0.775	1.706	1.403
Number with >1 mSv	6,277	407	1,275	3,186	1,384	1,480
Percent with >1 mSv [%]	65.1	4.6	15.4	39.4	65.4	68.1
Collective dose [person mSv]	13,885	4,068	5,113	7,196	3,651	3,454

## 4 Discussion

This study presented quantitative estimates of in-flight cosmic radiation doses of aircraft crews of a Japanese airline, including >8,000 cabin attendants and >2,100 pilots, over the COVID-19 pandemic period (2019–2022) for the first time. The annual doses of CAs were significantly affected by the pandemic. The number of the CAs who annually received >1 mSv remarkably decreased by >93% in the first year of the pandemic (2020) in comparison to the previous year, followed by a gradual recovery during the subsequent two years (~2022). These data indicate significant effects of the pandemic on the work of CAs, as previously reported (18–20).

In contrast, such changes were not observed with PL doses. This is partially attributable to an increase in the number of cargo flights during the pandemic period as observed worldwide (30–32), which contributed to an increase in flight opportunities only for pilots. In addition, according to information privately shared by the airline company, the stability of the PL doses was attributable to their efforts to reduce operational costs by introducing small aircrafts, which has continued over the pandemic period. To respond to the reduction in passengers, they attempted to maintain the operated flights for each route by promoting aircraft downsizing from the beginning of the pandemic. Consequently, while the flight opportunities of cabin attendants significantly decreased with the reduced number of passengers, those of pilots did not notably change because they were required to be on board regardless of aircraft size. This information is consistent with our previous findings which implied insignificant changes in the flight route distributions of Japanese travelers in the first year of the pandemic (2020) (21).

**Figure 4:**
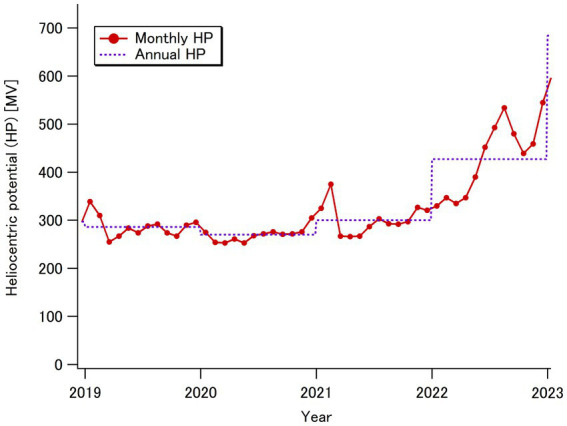
Changes in the monthly and annual average heliocentric potentials over the period from 2019 to 2022 (34).

The smaller PL doses in 2022 only in a higher dose range (>1.4 mSv per year) (Figure 3B) compared to those in 2019 could be partially explained by the change in solar activity shown in Figure 4. The heliocentric potential, which indicates the strength of solar modulation related to the cosmic radiation incidence to the Earth’s atmosphere (33), was relatively stable for the initial three years of this study (fiscal years 2019–2021), and notably increased in the fiscal year 2022 (34), which is considered to have reduced the cosmic radiation doses received only in long high-latitude flights. It should be noted that large solar flares, which can significantly affect cosmic radiation dose rates at aviation altitude (35), did not occur during the study period.

This study had some limitations, mainly due to the limited access to detailed records that could be used to identify individuals. While the annual doses of aircraft crews were calculated from the monthly determined route doses based on flight plans, real flights may have different times (speeds) and routes, including altitudes, owing to bad weather or traffic status. Potential errors due to such unexpected changes could not be quantified in this study because the authors were not allowed to obtain the precise flight-log data linked to individual crew members. In addition, we cannot discuss the effect of the pandemic on the cumulative doses of aircraft crews over two or more years because of the lack of information for personnel identification. In particular, missing PL doses during the initial period of the pandemic (2020–2021) have made this study incomplete, and it is desirable to present these critical data through a comprehensive analysis. We will continue further efforts to overcome these limitations for a deeper discussion on the recent changes in the cosmic radiation exposure of aircraft crews.

## Data Availability

The original contributions presented in the study are included in the article/supplementary material, further inquiries can be directed to the corresponding author.

## References

[1] Di Trolio R Di Lorenzo G Fumo B Ascierto PA. Cosmic radiation and cancer: is there a link? Future Oncol. (2015) 11:1123–35. doi: 10.2217/fon.15.29, PMID: 25804126

[2] Hayes K Megson D Doyle A O'Sullivan G. Occupational risk of organophosphates and other chemical and radiative exposure in the aircraft cabin: a systematic review. Sci Total Environ. (2021) 796:148742. doi: 10.1016/j.scitotenv.2021.148742, PMID: 34375198

[3] Scheibler C Toprani SM Mordukhovich I Schaefer M Staffa S Nagel ZD. Cancer risks from cosmic radiation exposure in flight: a review. Front Public Health. (2022) 10:947068. doi: 10.3389/fpubh.2022.947068, PMID: 36483259 PMC9723364

[4] Park S Lee GB Lee D Cha ES Han K Cho M. Cancer risk among air transportation industry workers in Korea: a national health registry-based study. BMC Public Health. (2024) 24:2435. doi: 10.1186/s12889-024-19904-w, PMID: 39244541 PMC11380205

[5] Bartlett DT. Radiation protection aspects of the cosmic radiation exposure of aircraft crew. Radiat Prot Dosim. (2004) 109:349–55. doi: 10.1093/rpd/nch311, PMID: 15273353

[6] International Commission on Radiological Protection (ICRP). 1990 recommendations of the international commission on radiological protection. ICRP Publ. 60. Ann. ICRP 21(1–3). (1991). 2053748

[7] International Commission on Radiological Protection (ICRP). The 2007 recommendations of the international commission on radiological protection. ICRP Publ. 103. Ann. ICRP 37(2–4), (2007). 10.1016/j.icrp.2007.10.003 18082557

[8] International commission on radiological protection (ICRP). Radiological protection from cosmic radiation in aviation. ICRP Publ. 132. Ann. ICRP 45(1) (2016). 10.1177/0146645316645449 27279360

[9] Friedberg W Copeland K Duke FE O'Brien K-III Darden EB. Radiation exposure during air travel: guidance provided by the Federal Aviation Administration for air carrier crews. Health Phys. (2000) 79:591–5. doi: 10.1097/00004032-200011000-00018, PMID: 11045535

[10] European Commission (EU). Council directive 96/29/EURATOM of 13 may 1996 laying down the basic safety standards for protection of the health of workers and the general public against the dangers arising from ionising radiation. Off J Eur Commun. (1996) 39:L159.

[11] Joint Aviation Authorities (JAA). Joint aviation requirements JAR-OPS 1 commercial air transportation (aeroplanes) subpart D—Operational procedures JAR-OPS-1.390 cosmic radiation. Tokyo: JAA. (2001).

[12] European Commission. Council directive 2013/59/ EURATOM of 5 December 2013 laying down basic safety standards for protection against the dangers arising from exposure to ionising radiation, and repealing directives 89/618/Euratom, 90/641/Euratom, 96/29/Euratom, 97/43/Euratom and 2003/122/Euratom. Brussels: EC. (2013).

[13] Yasuda H Sato T Yonehara H Kosako T Fujitaka K Sasaki Y. Management of cosmic radiation exposure for aircrew in Japan. Radiat Prot Dosim. (2011) 146:123–5. doi: 10.1093/rpd/ncr133 21613269

[14] Colgan PA Synnott H Fenton D. Individual and collective doses from cosmic radiation in Ireland. Radiat Prot Dosim. (2007) 123:426–34. doi: 10.1093/rpd/ncl527, PMID: 17223639

[15] Desmaris G. Cosmic radiation in aviation: radiological protection of air France aircraft crew. Ann ICRP. (2016) 45:64–74. doi: 10.1177/0146645316636009, PMID: 27044363

[16] Kubančák J Kyselová D Kovář I Hlaváčová M Langer R Strhársky I. Overview of aircrew exposure to cosmic radiation in the Czech Republic. Radiat Prot Dosim. (2019) 186:211–4. doi: 10.1093/rpd/ncz204, PMID: 31711207

[17] Lestaevel P Huet C Lejeune V Moreno C Villagrasa C Feuardent J. Cosmic radiation exposure of airline crews in France over the period 2015–2019. Radioprotection. (2023) 58:317–25. doi: 10.1051/radiopro/2023027

[18] Sharun K Tiwari R Natesan S Yatoo MI Malik YS Dhama K. International travel during the COVID-19 pandemic: implications and risks associated with ‘travel bubbles’. J Travel Med. (2020) 27:taaa184. doi: 10.1093/jtm/taaa184, PMID: 33009813 PMC7665670

[19] Steffen R Lautenschlager S Fehr J. Travel restrictions and lockdown during the COVID-19 pandemic—impact on notified infectious diseases in Switzerland. J Travel Med. (2020) 27:taaa180. doi: 10.1093/jtm/taaa180, PMID: 33152761 PMC7543597

[20] Dube K Nhamo G Chikodzi D. COVID-19 pandemic and prospects for recovery of the global aviation industry. J Air Transp Manag. (2021) 92:102022. doi: 10.1016/j.jairtraman.2021.102022, PMID: 36567961 PMC9759696

[21] Yasuda H Motoyama H Yajima K. Recent trends in cosmic radiation exposure onboard aircraft: effects of the COVID-19 pandemic on Japanese in-flight doses. Front Publ Health. (2025) 13:15543325. doi: 10.3389/fpubh.2025.1554332 PMC12043470 40313500

[22] National Institutes for Quantum and Radiological Science and Technology (QST). JISCARD: Japanese internet system for calculation of aviation route doses. Available online at: http://www.jiscard.jp/ (accessed on 1 July 2025).

[23] Sato T Yasuda H Niita K Endo A Sihver L. Development of PARMA: PHITS-based analytical radiation model in the atmosphere. Radiat Res. (2008) 170:244–59. doi: 10.1667/RR1094.1, PMID: 18666812

[24] Yasuda H Yajima K Sato T Takada M Nakamura T. Responses of selected neutron monitors to cosmic radiation at aviation altitudes. Health Phys. (2009) 96:655–60. doi: 10.1097/01.HP.0000345025.85844.97, PMID: 19430218

[25] Yasuda H Lee J Yajima K Hwang JA Sakai K. Measurement of cosmic-ray neutron dose onboard a polar route flight from New York to Seoul. Radiat Prot Dosim. (2011) 146:213–6. doi: 10.1093/rpd/ncr152, PMID: 21561941

[26] Yasuda H Yajima K. Verification of cosmic neutron doses in long-haul flights from Japan. Radiat Meas. (2018) 119:6–11. doi: 10.1016/j.radmeas.2018.08.016

[27] Yasuda H Kurita N Yajima K. Verification of estimated cosmic neutron intensities using a portable neutron monitoring system in Antarctica. Appl Sci. (2023) 13:3297. doi: 10.3390/app13053297

[28] Mares V Yasuda H. Aviation route doses calculated with EPCARD.Net and JISCARD EX. Radiat Meas. (2010) 45:1553–6. doi: 10.1016/j.radmeas.2010.06.015

[29] Bottollier-Depois JF Beck P Latocha M Mares V Matthiä D Rühm W. Comparison of codes assessing radiation exposure of aircraft crew due to galactic cosmic radiation. EURADOS Report 2012-03 (2012).

[30] Naseer S Khalid S Parveen S Abbass K Song H Achim MV. COVID-19 outbreak: impact on global economy. Front Public Health. (2023) 10:1009393. doi: 10.3389/fpubh.2022.1009393, PMID: 36793360 PMC9923118

[31] Budd L Ison S. Chapter 14–the impact of COVID-19 on air cargo logistics and supply chains In: eds. Zhang J, Hayashi Y. Transp amid pandemics. Amsterdam: Elsevier Ltd. (2022) 183–8. doi: 10.1016/C2020-0-04079-X

[32] Deng Y Zhang Y Wang K. An analysis of the Chinese scheduled freighter network during the first year of the COVID-19 pandemic. J Transp Geogr. (2022) 99:103298. doi: 10.1016/j.jtrangeo.2022.103298, PMID: 35125679 PMC8801321

[33] O'Brien K Felsberger E Kindl P. Application of the heliocentric potential to aircraft dosimetry. Radiat Prot Dosim. (2005) 116:336–42. doi: 10.1093/rpd/nci090, PMID: 16604656

[34] United States Department of Transportation Federal Aviation Administration (FAA). Heliocentric Potential. Available online at: https://www.faa.gov/data_research/research/med_humanfacs/aeromedical/radiobiology/heliocentric (accessed on 1 July 2025)

[35] Bütikofer R Flückiger EO Desorgher L Moser MR. The extreme solar cosmic ray particle event on 20 January 2005 and its influence on the radiation dose rate at aircraft altitude. Sci Total Environ. (2008) 391:177–83. doi: 10.1016/j.scitotenv.2007.10.021, PMID: 18031791

